# The complete mitochondrial genome sequence of an annual wild tobacco *Nicotiana attenuata*

**DOI:** 10.1080/23802359.2017.1407686

**Published:** 2017-12-07

**Authors:** Yiqing Xu, Fuquan Zhang, Kun Hu

**Affiliations:** a Department of Information Technology, PLA Army Engineering University, Nanjing, Jiangsu, China;; b College of Information Science and Technology, Nanjing Forestry University, Nanjing, Jiangsu, China;; c School of Computer Science and Engineering, Southeast University, Nanjing, Jiangsu, China

**Keywords:** *Nicotiana attenuata*, mitochondrial genome, phylogeny

## Abstract

*Nicotiana attenuata* (*N. attenuata*) is a species of wild tobacco, which is a predominant ecological model native to North America. Here, we first assembled the complete mitochondrial (mt) DNA sequence of *N. attenuata* into a circular genome of length 394,341 bp, including 40 protein-coding genes, 4 rRNA genes, and 23 tRNA genes. A neighbour-joining phylogenetic tree was constructed based on the complete mt genome of *N. attenuata* and other 27 plant species to assess their phylogenic relationship and evolution. The complete mt genome of *N. attenuata* would be useful for the further analyses of *Nicotiana* species.

*Nicotiana attenuata*, popularly known as coyote tobacco, is an annual herb that is native to western North America. Thanks largely to its diverse interactions with plenty of different plants, insects, and microorganisms in its native habitat, *N. attenuata* has been utilized as an ecological model species since 1994 (Baldwin et al. 1994). Additionally, *N. attenuata* is a species that can induce nicotine, volatiles and phenolics in response to damage (Dam et al. 2001).

Mitochondria encode the main manufactures of cellular ATP, and play vital roles in the regulation of cellular metabolism (Lei et al. 2013; Bi et al. 2016). In plant species, the mitochondrial (mt) genomes vary considerably in length, gene content and order (Richardson et al. 2013). Until now, there are only 274 complete plant mt genomes deposited in GenBank Organelle Genome Resources. In this study, we assembled the complete mt genome sequence of *N. attenuata*, which may provide more desirable information for better understanding the interactions of *N. attenuata* in its native environment and the nicotine biosynthetic pathway in *Nicotiana* species.

Sample of *N. attenuata* used for extraction was harvested from a native population in Washington Country, Utah (Geographic coordinate: 37° 16′ 48″ N, 113° 31′ 12″ W). The genomic DNA was isolated from late rosette-stage plants following the CTAB DNA extraction protocol (Bubner et al. 2004), and then deposited in Max Planck Institute for Chemical Ecology (Jena, Germany). In this study, the complete mt DNA sequence of *N. attenuata* was assembled into a gap-free and accurate genome of length 394,341 bp by Newbler v3.0 and MacVector v15.1.3, and then submitted to GenBank with the accession number MF579563. The GC content of the mt genome is 45.05%, a widespread value in most Asterids.

Using the annotation program MITOFY (Alverson et al. 2010), a total of 67 genes were identified in the mt genome of *N. attenuata*, including 40 protein-coding genes, 23 tRNA genes, and four rRNA genes. Among these, eight genes contain two copies (*rpl5*, *rps14*, *rrn26*, *trnM-CAT*, *trnfM-CAT*, *trnN-GTT*, *trnP-TGG*, and *trnE-TTC*). Additionally, 33 exons and 14 introns were identified in nine protein-coding genes (*rps3*, *cox2*, *nad1*, *nad2*, *nad5*, *nad7*, *rps10*, *ccmFc*, and *rpl2*). The analysis of codon usage shows that most of the mt protein-coding genes share the common start codon: ATG, except *rps10*, *cox1* and *rps4*, which use ACG (C to U RNA-editing) as start codon. Three types of stop codons are found in the mt protein-coding genes: TAA (20 genes: *atp1*, *atp8*, *cox1*, *cox2*, *nad1*, *nad2*, *nad3*, *nad4L*, *nad5*, *nad6*, *nad9*, *rpl2*, *rpl5*x2, *rpl10*, *rpl16*, *rps1*, *rps4*, *rps19*, and *sdh4*), TGA (13 genes: *atp6*, *ccmB*, *ccmC*, *ccmFc*, *ccmFn*, *cob*, *cox3*, *matR*, *nad4*, *rps10*, *rps12*, *rps13*, and *sdh3*), and TAG (seven genes: *atp4*, *atp9*, *mttB*, *nad7*, *rps3*, and *rps14*x2). A neighbour-joining phylogenetic tree was constructed using 28 plant mt genomes based on 23 conserved protein-coding genes, including 18 respiratory complex genes (*atp1*, *atp4*, *atp6*, *atp8*, *atp9*, *cob*, *cox1*, *cox2*, *cox3*, *nad1*, *nad2*, *nad3*, *nad4*, *nad4L*, *nad5*, *nad6*, *nad7*, and *nad9*), four cytochrome c biogenesis genes (*ccmB*, *ccmC*, *ccmFc*, and *ccmFn*) and one Maturase gene (*matR*). As shown in Figure 1, the phylogenetic tree exhibited that *N. attenuata* and *Nicotiana tabacum* are classified into one clade, and the clade of *Nicotiana* was evolutionarily close to the Solanaceae plant *Capsicum annuum*.

**Figure 1.**
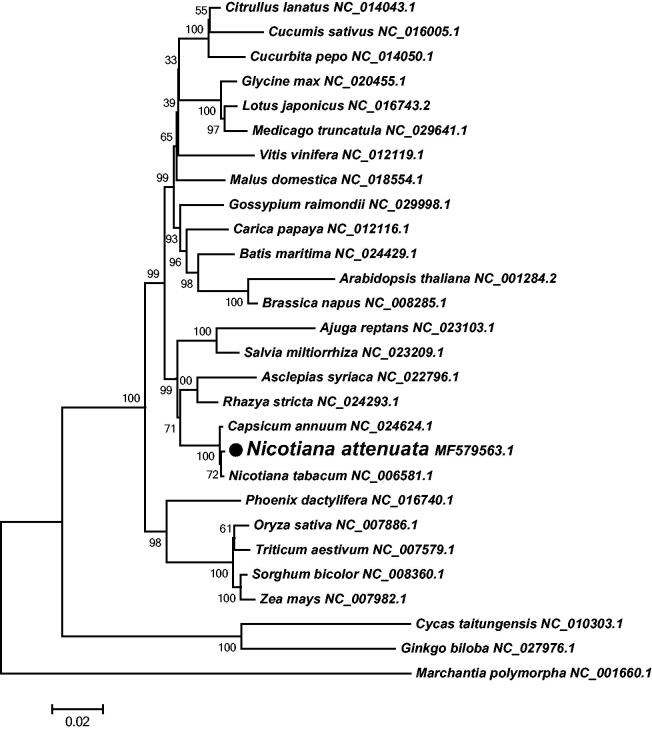
Neighbor-joining tree of 28 plant mt genomes based on 23 conserved protein-coding genes. Numbers on each node are bootstrap support values. Each genome’s accession number for tree construction is listed right to its scientific name.
